# Assessing nursing staff’s competences in mobility support in nursing-home care: development and psychometric testing of the Kinaesthetics Competence (KC) observation instrument

**DOI:** 10.1186/s12912-016-0185-z

**Published:** 2016-11-22

**Authors:** Heidrun Gattinger, Helena Leino-Kilpi, Virpi Hantikainen, Sascha Köpke, Stefan Ott, Beate Senn

**Affiliations:** 1 Department of Nursing Science, University of Turku, Turku, Finland; 2 Institute of Applied Nursing Science, FHS St. Gallen University of Applied Sciences, Rosenbergstrasse 59, 9001 St. Gallen, Switzerland; 3 Turku University Hospital, Turku, Finland; 4 Institute for Social Medicine and Epidemiology, Nursing Research Unit, University of Lübeck, Lübeck, Germany; 5 FHS St. Gallen University of Applied Sciences, St. Gallen, Switzerland; 6 Research Affiliate Sydney Nursing School, University of Sydney, Sydney, Australia

**Keywords:** Kinaesthetics, Mobility limitation, Educational measurement, Clinical competence, Nursing

## Abstract

**Background:**

Between 75 and 89% of residents living in long-term care facilities have limited mobility. Nurses as well as other licensed and unlicensed personnel directly involved in resident care are in a key position to promote and maintain the mobility of care-dependent persons. This requires a certain level of competence. Kinaesthetics is a training concept used to increase nursing staff’s interaction and movement support skills for assisting care-dependent persons in their daily activities. This study aims to develop and test an observation instrument for assessing nursing staff’s competences in kinaesthetics.

**Methods:**

The Kinaesthetics Competence (KC) observation instrument was developed between January and June 2015 based on a literature review, a concept analysis and expert meetings (18). The pilot instrument was evaluated with two expert panels (*n* = 5, *n* = 4) regarding content validity, usability and inter-rater agreement. Content validity was assessed by determining the content validity index (CVI). The final instrument was tested in a cross-sectional study in three nursing homes in the German-speaking part of Switzerland between July 2015 and February 2016. In this study nursing staff (*n* = 48) was filmed during mobilization situations. Based on this video data two observers independently assessed nursing staff’s competences in kinaesthetics with the KC observation instrument. Inter-rater reliability and inter-rater agreement was evaluated using the intra-class correlation coefficient (ICC) and percentage of agreement. Construct validity was assessed by a discriminating power analysis. Internal consistency was evaluated using Cronbach’s alpha coefficient and item analysis.

**Results:**

The final version of the KC observation instrument comprised of four domains (interaction, movement support of the person, nurses’ movement, environment) and 12 items. The final instrument showed an excellent content validity index of 1.0. Video sequences from 40 persons were analysed. Inter-rater reliability for the whole scale was good (ICC 0.73) and the percentage of inter-rater agreement was 53.6% on average. Cronbach’s alpha coefficient for the whole instrument was 0.97 and item-total correlations ranged from 0.76 to 0.90. The construct validity of the instrument was supported by a significant discrimination of the instrument between nursing staff with no or basic and with advanced kinaesthetics training for the total score and 3 of 4 subscales.

**Conclusions:**

The KC observation instrument showed good preliminary psychometric properties and can be used to assess nursing staff’s competences in mobility care based on the principles of kinaesthetics.

## Background

Between 75 and 89% of residents living in long-term care facilities have limited mobility [19, 30, 31]. Immobility is a major factor contributing to a reduced quality of life and preventable adverse events among older adults living in residential long-term care: increased incidences of urinary infections, pressure ulcers, contractures and falls, as well as a persistent decline in function and physical activity [5, 22]. Mobility problems affecting participation in social activities and independence, and therefore residents’ wellbeing [24]. Residents view mobility as a means of freedom, choice and independence and therefore identify mobility as being of central importance to quality of life and well-being [2]. Nurses as well as other licensed and unlicensed personnel, e.g. licensed practical nurses, nursing assistants or nursing aides, are involved in care tasks that include mobility support of care-dependent persons. In this paper we refer to this group as nursing staff. Nursing staff are in a key position to promote and maintain the functional abilities of care-dependent persons. The competences which nursing staff should have in order to perform interventions that promote mobility in activities of daily living (ADL), are emphasised in curricular guidelines and nursing standards [3, 4, 23].

Kinaesthetics is a training concept aimed to increase nursing staff’s interaction and movement support skills when assisting care-dependent persons in their daily activities [8, 18]. Kinaesthetics for nursing was developed by Hatch and Maietta [18] and further developed by the European Kinaesthetics Association [8, 20]. The theoretical base is found in behavioural cybernetics. From that viewpoint, human movement and behaviour are controlled through a self-governed, closed-loop control process. Human movement is the foundation for the way people experience and interact with other people and their surroundings. Furthermore, human beings perceive, learn and experience through bodily movement. Thus the kinaesthetic interaction is an important communication channel, and the core of kinaesthetics is the interaction between humans while moving. Kinaesthetics emphasises a person’s potential to learn new or different ways to move so as to overcome disabilities and gain more independence [18, 20]. Kinaesthetics courses are offered in registered and vocational nursing programs as well as for continuing education in health care institutions in German-speaking countries since 1990 and are currently trained in an increasing number of other European countries (e.g. Italy, Romania, Demark and Finland) [9]. The kinaesthetics program for nursing contains a continuing education starting with a basic course, an advanced training course and a peer-tutoring course, with a recommended length of between 24 and 42 training units [10]. In the basic courses participants learn the six kinaesthetics-dimensions: interaction, functional anatomy, human movement, human functions, effort and environment. Each of these dimensions offers a different perspective that can be used for a systematic analysis of human movement competence [18, 28]. During the advanced training course and the peer-tutoring course, participants gain a deeper understanding of human movement interaction based on this dimensions. There is the possibility to continue with a trainer education for kinaesthetics trainer levels 1, 2 and 3. Each kinaesthetics trainer program lasts for 1 year and contains between 280 and 380 training hours. Persons who successfully completed the kinaesthetics trainer level 1 are certified as kinaesthetics experts for clinical practice. A kinaesthetics trainer level 2 qualifies to carry out a kinaesthetics basic course and kinaesthetics trainer level 3 to teach in advanced training and peer-tutoring courses. The last level in the education system of the European Kinaesthetics Association is the trainer for trainers. Persons who passed this training are qualified to teach in the kinaesthetics trainer courses level 1, 2 and 3 [10].

In Switzerland, kinaesthetics training is offered by certified kinaesthetics trainers [11]. Basic and partly advanced training is incorporated in registered and licenced nursing education programs [25]. However, the evaluation of achieved competences as well as further education, e.g. peer-tutor or trainer education is a matter for each health care institution.

Assessing nursing staff’s competences after kinaesthetics training is important for making sure that learned principles were put to the best possible use in clinical practice and also for identifying areas for further development and educational needs. There has been much discussion in the literature about the type of instrument needed to assess competence or performance in health care, e.g. self-assessment, interviews or observations [6, 13, 16]. The method of assessment selected should be the most direct and relevant to the performance being assessed [16]. An observation method appears to be appropriate for assessing competence in kinaesthetics, as kinaesthetics training aims to develop nursing staff’s movement and interaction skills when assisting a care-dependent person with movement in activities of daily living, and this method allows performance in the workplace to be assessed. So far there is no valid and reliable observation instrument to assess nursing staff’s competences in kinaesthetics [15].

Therefore, the aim of this study was to develop and psychometrically [1] test the Kinaesthetics Competence (KC) observation instrument, an instrument to be used by kinaesthetics trainers or other health professionals who are familiar with kinaesthetics to assess the level of nursing staff’s competences in kinaesthetics.

## Methods

**Fig. 1:**
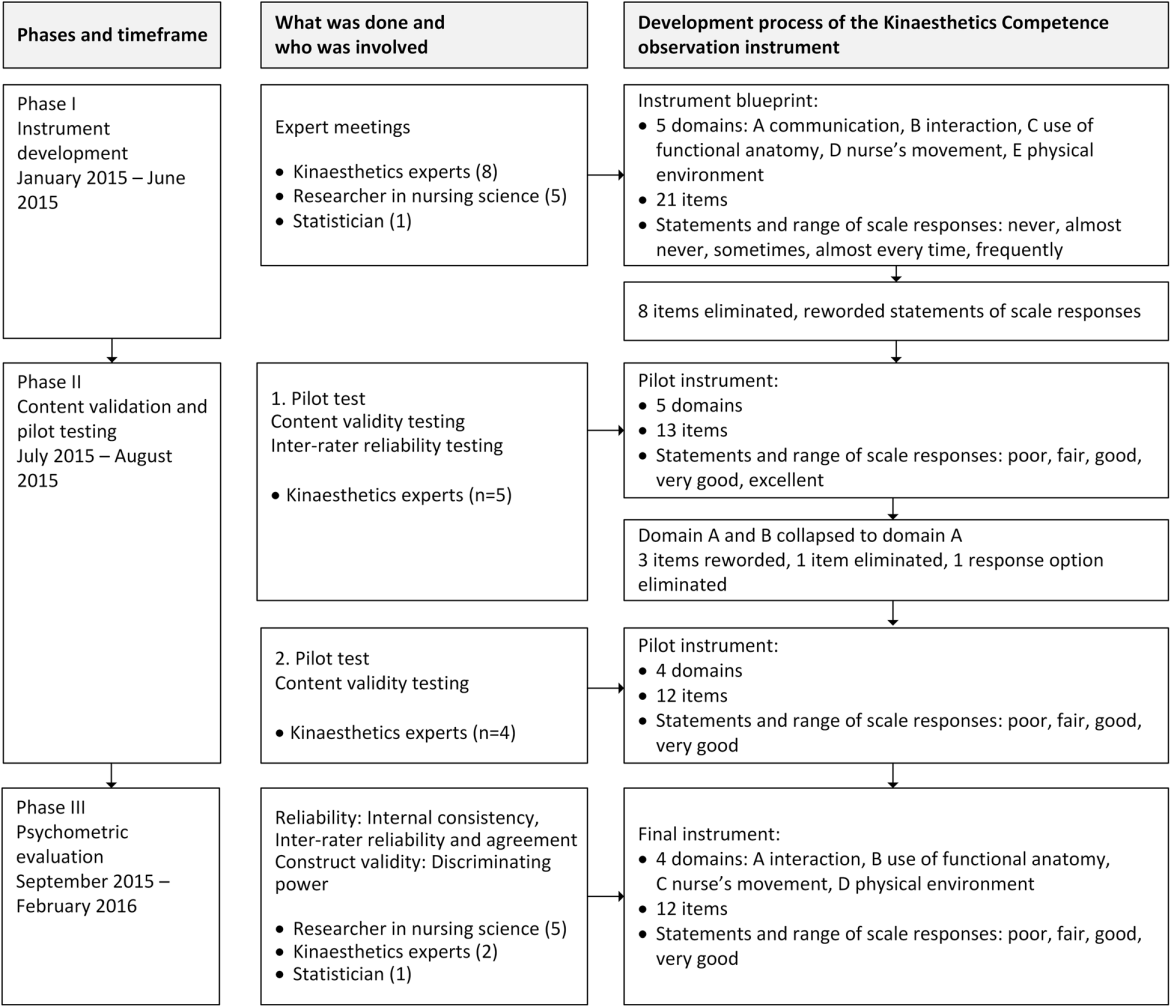
Development and validation process of the KC observation instrument. Visualization of phases and description about tasks, persons involved and the changes made in the KC observation instrument during the instrument development process

Three phases have been conducted to develop [29] and test [1] the instrument: 1) instrument development; 2) examining content validity and pilot testing; and 3) psychometric evaluation (Fig. 1).

### Phase I: instrument development

The construction of the instrument was begun by making a blueprint with an item pool and response scale based on a literature review of observational instruments used to assess nurses’ skills in patient mobilisation [15], a concept analysis of nurses’ competence in kinaesthetics [14] and on a first meeting of experts. The blueprint comprised 21 items in five domains (communication, interaction, use of functional anatomy, nurses’ movement, physical environment). In an iterative process that involved 13 experts and a statistician, the items and the response scale were refined between January 2015 and June 2015. During this time, a total of 18 face-to-face meetings with one or more of these experts were held. The expert group contained kinaesthetics experts (8, minimum a kinaesthetics trainer level 2) and researchers in nursing science (5, all with expertise in research and two with additional expertise in instrument development). The sociodemographic characteristics of the experts are shown in Table 1. Expert feedback was utilized to ensure that the instrument captures relevant items and was usable in clinical practice. After this process, the pilot instrument covered five domains with 13 items (Fig. 1).

**Table 1:** Experts’ sociodemographic characteristics involved in the expert meetings and the first and second pilot test

Characteristics	expert meetings (*n* = 13)	pilot test 1 (*n* = 5)	pilot test 2 (*n* = 4)
Age: mean (SD)			
In years	47.1 (9.456)	53.2 (3.899)	52.5 (1.732)
Nationality: n (%)			
Swiss	8 (61.5%)	5 (100%)	4 (100%)
German	2 (15.4%)		
Austrian	3 (23.1%)		
Profession: n (%)			
Nurse	5 (38.5%)	2 (40%)	1 (25%)
Researcher in nursing science	5 (38.5%)		1 (25%)
Physiotherapist	1 (7.7%)	1 (20%)	
Speech therapist	1 (7.7%)	1 (20%)	1 (25%)
Classical philologist	1 (7.7%)	1 (20%)	1 (25%)
Kinaesthetics training: n (%) ^a^			
Basic training	4 (30.8%)		
Trainer education level 2	1 (7.7%)	1 (20%)	1 (25%)
Trainer education level 3	4 (30.8%)	1 (20%)	1 (25%)
Train the trainer	4 (30.8%)	3 (60%)	2 (50%)
Work setting as kinaesthetics trainer: n (%) ^b^			
Long-term institutional care	9 (69.2%)	5 (100%)	4 (100%)
Hospital care	4 (30.8%)	2 (40%)	2 (75%)
Home care	4 (30.8%)	2 (40%)	3 (25%)
Working experience with kinaesthetics: mean (SD)			
In years	14.7 (9.050)	18.2 (10.085)	16.8 (7.136)

^a^According the European Kinaesthetics Association [10]; ^b^Double entries possible (added up to more than 100%)

### Phase II: content validity and pilot testing

The pilot instrument was first tested in order to investigate usability, e.g. time required for administering the assessment, and inter-rater agreement in July 2015. For this purpose two nurses with different kinaesthetics training levels from the participating nursing homes (see Phase III) were filmed in three different mobilisation situations, e.g. a transfer from bed to wheelchair or a transfer from wheelchair to chair. An expert panel of five kinaesthetics trainer (Table 1) administered the pilot instrument on the video data. The experts were asked (1) to familiarise themselves with the pilot instrument, (2) to watch the video sequences of each person two to three times in order to focus on the domains separately (e.g. first focusing on interaction and movement support of the person and then on movement of the nurse and physical environment), (3) to rate the persons’ competences on the pilot instrument and (4) to note how long each assessment lasted. The experts were also involved in the content validity test of the instrument and were asked to rate the relevance of each item included in the pilot instrument on a content validity index (CVI) rating form [26]. The question to experts was: “Please rate the extent to which this item is relevant to assess nursing staff’s competences in kinaesthetics” rated on a 4-point Likert scale (1 = not relevant, 2 = somewhat relevant, 3 = quite relevant, 4 = highly relevant). All participants were further asked if the instructions and the items are understandable and clear and, if not, how they would revise the issue of concern. Two last questions asked if there is something missing and if there are further comments / suggestions to improve the pilot instrument.

The second pilot test with four experts took place in August 2015. The sociodemographic characteristics of the experts are described in Table 1. The aim was to test the content validity and usability of the revised instrument. Therefore, the experts were asked to (1) rate the relevance of the items on a 4-point Likert scale as described above, (2) evaluate the clarity of the items and (3) the clarity of the instrument instructions.

### Phase III: psychometric evaluation

In the third phase, to explore the psychometric properties of the instrument, a cross-sectional study has been conducted. Data was collected between September and November 2015 from the target population of nursing staff (*n* = 214) working in three medium-sized nursing homes in the German-speaking part of Switzerland. A consecutive purposive sample [7] was recruited based on the following inclusion criteria: 1) nursing staff (i.e. registered nurses, licensed practical nurses, assistant nurses and student nurses) working in direct care, 2) nursing staff with no or only basic and advanced training in kinaesthetics (approximately equal proportions) and 3) their informed consent.

The recruitment process of the nursing home residents was as follows: The chief nurse of each nursing home assessed the residents for eligibility based on the following inclusion criteria: 1) slightly or very limited mobility or completely immobile assessed with the item “mobility” on the Braden scale (score between 1 and 3) [17], 2) able to give informed consent and 3) voluntary participation. Eligible nursing home residents were then visited by the researcher (HG) in order to acquire informed consent.

Study participants were observed during mobilization situations and these situations were recorded with a video camera (Canon HD Camcorder HG10). The recordings were conducted in the residents’ rooms or the living rooms by the researcher (HG). Nursing staff’s competence in kinaesthetics was rated based on the video recordings. In order to get a meaningful picture of the competences, 2–3 video sequences for each person were selected based on the following criteria: 1) video sequence shows a good view of the person of interest (in case two persons were involved), 2) different mobilization situations (e.g. helping resident out of bed or assistance with ambulation), 3) different residents are assisted.

In order to assess inter-rater reliability, four observers (HG, VP and two kinaesthetics experts) were involved in the data analysis. Before each person carried out the data analysis independently, a 4-h training session was conducted.

### Ethical considerations

At all phases, this study followed the principles of the Helsinki Declaration [32]. Ethical approval was obtained from the ethics committee in charge (EKSG 14/009L) and permission to conduct the study was obtained from the chiefs of the nursing homes. Nursing staff and nursing home residents were informed personally by the researcher (HG) and in writing that participation was voluntary, about their right to withdraw at any time and that their research records would remain anonymous, and that all information would be treated confidentially. In the video data the faces of the participants were visible and participants had been informed about this. Safe storing of the video data was assured and only a small number of experts involved in this study was allowed to see them for data analysis. After finishing the study, the video data were deleted. Both nursing staff and residents involved in the pilot test and in the main study gave their written informed consent.

### Data analysis

All statistical data analyses were performed using SPSS 22 (IBM Corp., 2013). Descriptive statistics to analyse the data include frequencies, means and standard deviation.

The content validity index (CVI) of individual items (I-CVI) and the entire scale (S-CVI) was calculated for both rounds of pilot-testing and followed the guidelines recommended by Polit and Beck [26]. The I-CVI for each item was calculated by summarizing the number of experts giving a 3 (quite relevant) or 4 (highly relevant) rating divided by the total number of experts who completed the test. The S-CVI universal agreement was calculated by summarizing items that achieved a rating of 3 or 4 by the experts divided by the total number of items [26]. According to Polit and Beck [26], the I-CVI should be no lower than 0.78 when there are six or more judges and 1.00 in case of five or fewer judges. For the S-CVI, a value of 0.90 or higher is recommended.

The internal consistency of the instrument was assessed by calculating Cronbach’s Alpha at subscale and total scale level. A Cronbach’s alpha higher than 0.80 was considered as satisfactory [27]. Item analysis was performed by computing the corrected item-total correlation for the items in the subscales. Item-total correlations of at least 0.20 were regarded as acceptable [27].

For inter-rater reliability the intraclass correlation (ICC) was calculated for each item and for the total score by using a one-way random effects model. Reliability coefficient values below 0.40 were considered poor, values between 0.41 and 0.75 fair to good and values greater than 0.75 excellent [27]. Additionally, the percentage of agreement as recommended by Kottner et al. [21] is reported. Percentage of agreement was defined by the number of times the observer agreed to the same response divided by the number of observations.

The construct validity of the instrument was assessed by a discriminating power (known-group technique) analysis [27]. To study the discriminating power of the instrument, two groups with a theoretically expected difference in kinaesthetics competence were predefined: nursing staff with no or basic kinaesthetics training vs. nursing staff with advanced kinaesthetics training. Wilcoxon rank-sum tests were used to calculate whether the scores of two independent groups have a similar ranked distribution for the mean subscale scores and the total score of the predefined groups. A significance level of 0.05 using a one-tailed test was applied. Discriminant evidence was provided if nursing staff with higher levels of training obtained significantly higher KC observation instrument scores.

## Results

### Phase II: content validity and pilot testing

During the first pilot test five experts independently rated the competence of two persons. Person 1 had no kinaesthetics training while person 2 had passed advanced kinaesthetics training. The overall mean percentage of agreement was 41.9% and varied between 15.4 and 61.5%. The percentage agreement across all pairs of observer are displayed in Table 2. The time the experts needed for the assessment was on average 39.8 and 36.5 min, respectively. The content validity of the pilot instrument had a scale CVI of 0.98. Experts’ assessment of 13 items showed an excellent CVI of 1.0 for 12 items and a CVI of 0.8 for one item.

**Table 2:** Inter-rater agreement of ten observer pairs based on two assessments with the KC observation instrument during the first pilot test

Participant	Observer pairs										mean
	O1 + O2	O1 + O3	O1 + O4	O1 + O5	O2 + O3	O2 + O4	O2 + O5	O3 + O4	O3 + O5	O4 + O5	
Person 1	61.5%	46.2%	53.9%	15.4%	53.9%	61.5%	7.7%	61.5%	7.7%	0%	36.9%
Person 2	53.9%	61.5%	38.5%	61.5%	53.9%	61.5%	30.8%	38.5%	38.5%	30.8%	46.9%
mean	57.7%	53.9%	46.2%	38.5%	53.9%	61.5%	19.2%	50%	23.1%	15.4%	41.9%

*O1* observer one, *O2* observer two, *O3* observer three, *O4* observer four, *O5* observer five

Based on the experts’ feedback, the domains “communication” and “interaction” were integrated with the domain “interaction”, one item was deleted and three items were re-worded. Based on the inter-rater agreement testing, the response options were reduced from five to four categories.

In the second pilot test, the revised instrument had a scale CVI of 1.0 based on four experts’ ratings (Table 3). There were no suggested changes to the content of this version. The framework of the final instrument is shown in Table 3.

**Table 3:** Structure of the final KC observation instrument and its second pilot test’s item content validity (I-CVI) results

Domains		No of items	Items	I-CVI
A	Interaction	3	A1 Communication	1.0
			A2 Mutual guiding	1.0
			A3 Time, space, effort	1.0
B	Movement support of the person	5	B1 Use of persons’ movement possibilities	1.0
			B2 Move body parts individually	1.0
			B3 Weight shift in direction of bone structure	1.0
			B4 Weight control with limbs	1.0
			B5 Weight shift using a supportive surface	1.0
C	Nurses’ movement	3	C1 Use of own movement possibilities	1.0
			C2 Adaptation of effort	1.0
			C3 Weight shift onto bone structure	1.0
D	Environment	1	D1 Adjustment of environment	1.0

### Description of the final KC observation instrument

The final Kinaesthetics Competence observation instrument consist of four domains and 12 items (Table 3). The scale response options were four categories: poor, fair, good, very good. Single items scored from 1 to 4. Criteria for the assessment of the four levels and the corresponding score is shown in Table 4. In cases where an item is not observable, an additional option of “not observable” scored with 0 is available. Mean scores are calculated for the KC observation instrument subscales (range 1–4). Non-observable items are omitted from the final calculation. The total score is calculated by adding up the subscales’ mean scores (range 4–16).

**Table 4:** Assessment categories of the KC observation instrument, the criteria and corresponding score

Category	Criteria	Score
poor	Lack of awareness or limited capability - significant area(s) of weakness or concern in communication/interaction - no/very limited understanding of functional movement in daily activities - little adaptation of own movement - no/inappropriate adaptation of environment	1
fair	Developing - beginning adaptation in communication/interaction - beginning understanding of functional movement in daily activities - beginning adaptation of own movement - beginning adaptation of environment	2
good	Capable - good adaptation in communication/interaction - good understanding of functional movement in daily activities - good adaptation of own movement - good adaptation of environment	3
very good	Best practice - very good adaptation in communication/interaction - very good understanding of functional movement in daily activities - very good adaptation of own movement - very good adaptation of environment	4

### Phase III: psychometric evaluation

#### Sample characteristics of nursing staff and nursing home residents

Of the nursing staff 48 persons were included in the study. Out of this sample eight persons could not be assessed as they were filmed in only one mobilisation situation or the mobilisation sequences did not allow a reliable rating (e.g. situations involving three persons). Consequently, 40 persons could be included in the final analysis. Participants had an average age of 39.1 years (SD = 14.421). 37.5% (*n* = 15) had no or only a basic qualification in kinaesthetics and 62.5% (*n* = 25) had completed additional training such as an advanced course or a kinaesthetics trainer training. For further details, see Table 5.

**Table 5:** Nursing staff’s (*n* = 40) and residents’ (*n* = 31) sociodemographic characteristics

Characteristics nursing staff	mean (SD)	n (%)
Age		
In years	39.1 (14.421)	
Experience in long-term care		
In years	10.0 (8.429)	
Working in the current institution		
In years	6.7 (6.516)	
Gender		
Female		32 (80%)
Male		8 (20%)
Educational level		
Registered nurse (Diploma, Bachelor)		14 (35%)
Licensed practical nurse (3 years training)		7 (17.5%)
Assistant nurse (up to 2 years training)		16 (40%)
Student nurse		2 (5%)
Missing information		1 (2.5%)
Kinaesthetics training ^a^		
None		4 (10%)
Basic training course		11 (27.5%)
Advanced training course		16 (40%)
Peer tutoring training		5 (12.5%)
Trainer (level 1–3) and train the trainer		4 (10%)
Characteristics residents		
Age		
In years	76.9 (13.928)	
Living in resident home		
In years	4.2 (3.896)	
Gender		
Female		20 (64.5%)
Male		11 (35.5%)
Activity ^b^		
Bedridden		0
Wheelchair-bound		17 (54.8%)
Walking short distances		11 (35.5%)
Regular walking		3 (9.7%)
Mobility ^b^		
Completely immobilised		1 (3.2%)
Severely reduced mobility		12 (38.7%)
Slightly reduced mobility		18 (58.1%)
No limitations in mobility		0

^a^according the European Kinaesthetics Association [10]; ^b^Item on the Braden Scale [17]

Thirty-one residents with a mean age of 76.9 years were included in the study. More than half of these residents (54.8%) was wheelchair-bound and 38.7% had severely reduced mobility. For further details, see Table 5.

#### Descriptive results of the instrument

Across the single observation criteria 10 to 18% of the nursing staff received very good, 30 to 63% good and 23 to 55% fair evaluations. Few participants (3–5%) were judged to perform poorly. For the domains nurses’ movement and environment, all participants received fair to very good evaluations (Table 6).

**Table 6:** Psychometric testing of the KC observation instrument: results to scale descriptives, internal consistency, inter-rater reliability and agreement

	Scale descriptives n (%)^a^								
Domains and items of the instrument	poor	fair	good	very good	Cronbach’s α for subscale	Item-total correlation for subscale ^b^	ICC	95% CI	Agreement
*KC observation instrument*					0.97		0.73	0.50–0.86	53.6%
*A Subscale Interaction*					0.90				
A1 Communication	1 (3)	15 (38)	19 (48)	5 (13)		0.781	0.70	0.43–0.84	52.5%
A2 Mutual guiding	2 (5)	16 (40)	18 (45)	4 (10)		0.761	0.59	0.24–0.79	47.5%
A3 Time, space & effort	2 (5)	12 (30)	21 (53)	5 (13)		0.847	0.59	0.23–0.78	47.5%
*B Subscale Movement support of the person*					0.93				
B1 Use of persons’ movement possibilities	1 (5)	20 (50)	14 (35)	4 (10)		0.890	0.74	0.50–0.86	57.5%
B2 Move body parts individually	2 (5)	20 (50)	14 (35)	4 (10)		0.784	0.56	0.18–0.77	50%
B3 Weight shift in direction of bone structure	1 (3)	19 (48)	16 (40)	4 (10)		0.803	0.72	0.47–0.85	50%
B4 Weight control with limbs	2 (5)	22 (55)	12 (30)	4 (10)		0.782	0.54	0.13–0.75	45%
B5 Weight shift using a supportive surface	1 (3)	15 (38)	19 (48)	5 (13)		0.828	0.75	0.53–0.87	60%
*C Subscale Nurses’ movement*					0.94				
C1 Use of own movement possibilities	0	16 (40)	17 (43)	7 (18)		0.838	0.74	0.52–0.86	67.5%
C2 Adaptation of effort	0	9 (23)	25 (63)	6 (15)		0.897	0.62	0.28–0.80	55%
C3 Weight shift onto bone structure	0	11 (28)	24 (60)	5 (13)		0.904	0.61	0.26–0.79	57.5%
*D Item Environment*									
D1 Adjustment of environment	0	18 (45)	17 (43)	5 (13)			0.69	0.42–0.84	55%

^a^distribution of nursing staff’s assessment over the single items; ^b^correlation between the item score and the subscale score; *ICC* intraclass correlation coefficient

#### Internal consistency

Cronbach’s Alpha was 0.97 for the total KC observation instrument scale. For the subscales Cronbach’s Alpha was between 0.90 and 0.94 (Table 6). Regarding the item analysis, all of the items in subscale A interaction, B movement support of the person and C nurses’ movement were higher than the usual criteria set (*r* ≥ 0.20) [27].

#### Inter-rater reliability and agreement

Three persons with kinaesthetics trainer training (*n* = 3) and the first author (HG) independently assessed each 20 of the participants. The first author has passed an advanced training course in kinaesthetics and became very familiar with the concept of kinaesthetics during the whole project. The average time needed to analyse the two to three video sequences per participant was 14.7 min (ranged from 6 to 25 min). The results from the inter-rater reliability and the percentage of agreement analysis are summarized in Table 6. The ICC of the KC observation instrument scores for 40 participants was 0.73, for the single items ICC ranged between 0.54 and 0.75. Percentage of agreement was on average 53.6% and ranged from 45 and 67.5%.

#### Discriminating power

The results on discriminating power demonstrated significant differences between the two predefined groups in the total score and in three of four subscales, as shown in Table 7. Nursing staff with advanced kinaesthetics training had higher scores than nursing staff with no or basic kinaesthetics training at the subscale movement support of the person (2.75 vs. 2.25, *p* = 0.011) and nurses’ movement (3.04 vs. 2.53, *p* = 0.007), as well as for the item adjustment of environment (2.88 vs. 2.33, *p* = 0.012). Nursing staff with advanced kinaesthetics training had a higher score at the total scale level (11.46 vs. 9.61, *p* = 0.009), but the two groups did not significantly differ between the subscale interaction (2.79 vs. 2.49, *p* = 0.1).

**Table 7:** Discriminating power of the KC observation instrument

	Mean (SD)					
Subscale (1–4) and total (4–16) score	Total sample (*n* = 40)	Nursing staff without or with basic kinaesthetics training (*n* = 15)	Nursing staff with advanced kinaesthetics training (*n* = 25)	W	Z	P
Interaction	2.68 (0.673)	2.49 (0.469)	2.79 (0.757)	262	−1.298	0.1
Movement support of the person	2.57 (0.651)	2.25 (0.437)	2.75 (0.693)	262.5	−2.287	0.011^a^
Nurses’ movement	2.85 (0.622)	2.53 (0.451)	3.04 (0.641)	224	−2.448	0.007^a^
Adjustment of environment	2.68 (0.694)	2.33 (0.488)	2.88 (0.726)	230	−2.376	0.012^a^
Total score	10.77 (2.439)	9.61 (1.455)	11.46 (2.664)	223	−2.362	0.009^a^

*SD* standard deviation, *W* value Wilcoxon rank-sum test; ^a^significant on a one-tailed test level of 5%

## Discussion

In this study the Kinaesthetics Competence observation instrument for assessing nursing staff’s practical competences in mobility care based on the principles of kinaesthetics has been developed and tested.

### Content and discriminant validity

The content of the instrument was established in a thorough process of several expert meetings and two pilot tests. The judgement of experts on the final version of the instrument reached unanimous agreement regarding the relevance of all items included (S-CVI of 1.0). The scale descriptives showed that between 3 and 5% of the participants achieved poor ratings and that no poor ratings occurred for the items C1 (use of own movement possibilities), C2 (adaptation of effort), C3 (weight shift onto bone structure), and D1 (adjustment of environment). Between 10 and 18% of the participants achieved best scores over all items. Thus, neither a floor nor a strong ceiling effect has been observed. Testing discriminant validity demonstrates a significant difference between the two groups: nursing staff without kinaesthetics training or basic course had lower total scores and lower scores for the sub-scales movement support of the person, nurses’ movement and environment than nursing staff with advanced training in kinaesthetics. This means that the instrument is able to discriminate between different training levels and this supports the construct validity of the instrument [27]. However, for the subscale interaction, the difference between the two groups was not significant. Nursing staff without kinaesthetics training or a basic course may have achieved a higher score in this subscale because of other factors, e.g. learning from role-models.

### Internal consistency and inter-rater reliability

The analysis of the internal consistency evaluation showed that the instrument presents homogeneity among its items, indicating that the items are measuring the same underlying concept [27]. However, the high Cronbach’s alpha for the entire scale (0.97) and the high corrected item-total correlations (>0.70) within the sub-scales may suggest that there are item redundancies, meaning that items are in essence asking the same question in a slightly different way [12].

The reliability of an assessment instrument is based on interaction between the instrument, the sample and the situation [27]. In the current study we assessed the agreement between two observers, rating nursing staff with different kinaesthetics training levels in different mobilization situations regarding their competences in kinaesthetics. The average inter-rater agreement improved from 41.9 to 53.6% following the modifications made after the pilot test. The inter-rater reliability for the entire score (ICC 0.73) as well as for most single items was good. However, the four items A2 (mutual guiding), A3 (time, space & effort), B2 (move body parts individually) and B4 (weight control with limbs) showed fair inter-rater reliability results (ICC between 0.41 and 0.60). The confidence intervals for some items were quite large and the percentage of agreement for the three items A2 (mutual guiding), A3 (time, space & effort), and B4 (weight control with limbs) was less than 50%. A wide variance of scores between two raters might be due to either too generous or lenient assessment of participants, which could lead to a measurement error. Despite that, raters’ understanding and interpretation of each item could have differed, leading to this discrepancy. Thus raters’ competency and their level of training regarding the assessment process must be taken into consideration.

It would be beneficial for future research to also incorporate extensive training and detailed guidelines for observers with regard to assessment of the items in order to improve agreement and consistency between observers [29].

### Strengths and limitations of the study

The strengths of this study are that we developed this instrument in a group that included nursing researchers and experts with kinaesthetics trainer experience in different nursing care settings. Besides the panel’s broad experience, participants demonstrated a high level of commitment to this study. Another strength is that we tested the instrument in clinical practice and not in a laboratory setting, e.g. with a simulated patient [15]. A limitation of this study is that we had included only nursing homes where the concept of kinaesthetics is well implemented. Our sample may not therefore have included enough nursing staff with poor competences in kinaesthetics.

Another limitation is related to the use of the instrument: As we used the KC observation instrument in video data, information about the context, e.g. the mobility limitations of the resident, were lacking. If the instrument is used in future video observations, this aspect should be addresses by providing the observers with detailed information about the resident’s mobility limitations.

## Conclusions

### Practical implications

The KC observation instrument is a valid and reliable instrument that can be used to assess nursing staff’s competences in mobility care based on the principles of kinaesthetics in residential long-term care facilities. The results indicate that the KC observation instrument can make a valuable contribution to high-quality mobility care by assessing nursing staff’s competences from the perspectives of kinaesthetics and it can also be used to evaluate kinaesthetics training programmes. Furthermore, results from using the KC observation instrument to evaluate competence in kinaesthetics may assist nursing staff in their own development by identifying strengths as well as areas that need to be improved.

### Suggestions for further research

In the present study, we started to collect a body of evidence by researching content and discriminant validity. However, validity testing should be continued, e.g. by testing criterion validity. Test-retest reliability should also be determined in a future study. As the Cronbach’s alpha coefficient of the entire scale was high, further testing (i.e. factor analysis) would be beneficial in order to support a decision about item reduction, taking into account that in a factor analysis the domain environment, including only one item, may result in a low factor loading. Furthermore, wider use of the KC observation instrument, e.g. in hospital or home care settings, could strengthen the current results. Further studies should include a larger and diverse sample, especially including nursing staff without or with only a basic kinaesthetics training.

As we tested the instrument on video data, its use in direct observation has yet to be determined. One of the benefits of direct observation is the straightforward application without any need for equipment. In future studies, the testing of the KC observation instrument should be repeated using the format of direct observation in order to determine if the strategy used in this study leads to similar results.

## Abbreviations

CI: Confidence interval
CVI: Content validity index
ICC: Intraclass correlation coefficient
I-CVI: Item content validity index
KC: Kinaesthetics competence
S-CVI: Scale content validity index
SD: Standard deviation
